# Contemporary Multi-modality Imaging of Prosthetic Aortic Valves

**DOI:** 10.31083/RCM25339

**Published:** 2025-01-14

**Authors:** Bryan Q Abadie, Tom Kai Ming Wang

**Affiliations:** ^1^Section of Cardiovascular Imaging, Department of Cardiovascular Medicine, Cleveland Clinic, Cleveland, OH 44195, USA

**Keywords:** prosthetic valve dysfunction, multimodality imaging, echocardiography, cardiac computed tomography

## Abstract

With the aging of the general population and the rise in surgical and transcatheter aortic valve replacement, there will be an increase in the prevalence of prosthetic aortic valves. Patients with prosthetic aortic valves can develop a wide range of unique pathologies compared to the general population. Accurate diagnosis is necessary in this population to generate a comprehensive treatment plan. Transthoracic echocardiography is often insufficient alone to diagnose many prosthetic valve pathologies. The integration of many imaging modalities, including transthoracic echocardiography, transesophageal echocardiography, cardiac computed tomography, cardiac magnetic resonance imaging, and nuclear imaging, is necessary to care for patients with prosthetic valves. The purpose of this review is to describe the strengths, limitations, and contemporary use of the different imaging modalities necessary to diagnose prosthetic valve dysfunction.

## 1. Introduction

Aortic valve replacement, surgical or transcatheter, is recommended for treating symptomatic severe aortic valve stenosis (AS) and/or regurgitation (AR), or is considered when there is significant left ventricular dysfunction, dilation, or abnormal exercise stress test capacity or hemodynamic response in asymptomatic patients [1, 2]. Over the last two decades, there has been a significant increase in the volume of aortic valve replacement procedures [3]. Patients with prosthetic aortic valves can develop a myriad of complications and therefore need serial monitoring. Transthoracic echocardiography (TTE) continues to be the first-line imaging modality for the interrogation of prosthetic aortic valves; however, multimodality imaging has become vital to the care of these patients. The purpose of this article is to review how TTE, transesophageal echocardiography (TEE), cardiac computed tomography (CT), nuclear imaging, and magnetic resonance imaging (CMR) can be integrated to diagnose prosthetic aortic valve pathology and guide treatment (Table 1).

**Table 1.** **Indications, strengths, and limitations of transesophageal echocardiography, computed tomography, nuclear imaging, and cardiac magnetic resonance imaging**.

Modality	Indications	Strengths	Limitations
Transthoracic echocardiography	Routine monitoring of prosthetic valves	Widely available	Limited by poor acoustic windows and poor Doppler alignment
	First-line assessment for prosthetic valve dysfunction	Non-invasive	Reverberation and shadowing from prosthetic materials can limit visualization
	Assess valve and ventricular hemodynamics with exercise	High temporal resolution	
		Hemodynamic assessment of valves	
Transesophageal echocardiography	Assessment of valve morphology and gradients where there are poor acoustic windows on transthoracic echocardiography	High-spatial and temporal resolution	Limited by poor acoustic windows and poor Doppler alignment
	Determine mechanism of aortic regurgitation when unclear by TTE	Hemodynamic assessment of valves	Reverberation and shadowing from prosthetic materials can limit visualization
	Assessment of vegetations/masses when unclear by TTE	3D assessment for better anatomic definition	Typically requires sedation or anesthesia
Cardiac computed tomography	Assessment of leaflet motion (*e.g.*, elevated gradients, restricted motion on echocardiography)	High-spatial resolution	Lower temporal resolution compared to echocardiography
	Differentiation of thrombus versus pannus	Complete 3D anatomic assessment	Blooming artifact may limit interpretation of images
	Diagnosis and monitoring of hypoattenuating leaflet thickening		Lack of hemodynamic assessment
	Evaluation of invasive endocarditis (*e.g.*, paravalvular abscess, pseudoaneurysm)		Radiation
	Pre-procedural planning for Valve-in-Valve TAVR		Frequently requires iodinated contrast which limits use in patients with renal impairment or contrast allergies
Nuclear imaging	Aid in diagnosis of equivocal cases of prosthetic valve endocarditis	Sensitive for the diagnosis of endocarditis	Lacks Specificity
			Radiation
Cardiac magnetic resonance imaging	Chamber size and quantification to determine timing of intervention	Gold-standard for quantification of ventricular size and function	Limited spatial and temporal resolution
	Quantification of aortic regurgitation when equivocal by echocardiography	Hemodynamic assessment of valves	May underestimate aortic valve velocity/gradients compared to echocardiography
		Results may have prognostic significance	Imaging can be compromised by artifact from prosthetic materials or cardiac implantable devices
			Limited by patient motion and arrythmia
			Challenging in claustrophobic patients or patients unable to perform breath holds

TTE, transthoracic echocardiography; TAVR, transcatheter aortic valve replacement; 3D, 3-dimensional.

## 2. Types of Prosthetic Aortic Valves

Prosthetic aortic valves can be classified into four distinct categories: mechanical, bioprosthetic, homograft, and autograft [4]. The primary focus of this article will be on mechanical and bioprosthetic aortic valve.

The design of mechanical aortic valves has significantly changed over the last 50 years, progressing from ball-in-cage to single leaflet to the modern standard, bileaflet. Bileaflet mechanical valves consist of two leaflets and a metal ring, typically made from stainless steel, titanium, or pyrolytic carbon, wrapped by a knitted fabric [5, 6]. Because of their great long term durability, mechanical valves are preferentially placed in younger patients; the disadvantage of mechanical valves is the need for anticoagulation, specifically with vitamin K antagonists [7, 8].

Bioprosthetic valves derive primarily from bovine pericardium, porcine pericardium, or porcine aortic valves. These valves are chemically treated to promote durability and mounted on either a stented or stentless frame [6]. The primary advantage of bioprosthetic valves is their safety in the absence of systemic anticoagulation, only requiring low-dose aspirin. Although more recent data suggests improvements in long-term durability, bioprosthetic valves have significantly shorter lifespans than mechanical valves, generally lasting ~10–15 years. Importantly, bioprosthetic valves in younger patients degenerate faster than older patients [9, 10]. Whereas mechanical valves can only be placed surgically, bioprosthetic valves can be implanted surgically or percutaneously [11].

Homografts are valves explanted from cadavers and subsequently implanted in patients. The primary advantage of homografts is the lower risk of endocarditis and therefore they are frequently implanted in patients with a prior history of prosthetic valve endocarditis or at higher risk of developing a future infection [12].

There are several techniques to replace the aortic valve with autologous tissue. In the Ross procedure, an autologous pulmonic valve is positioned into the aortic valve position, with a homograft then placed in the pulmonic valve position. In the Ozaki procedure, the aortic valve is replicated using a patient’s pericardial tissue [13].

## 3. Prosthetic Valve Dysfunction – Clinical Perspectives

The American Society of Echocardiography (ASE), in conjunction with the Society for Cardiovascular Magnetic Resonance Imaging and the Society of Cardiovascular Computed Tomography, recently published formal guidelines on the evaluation of prosthetic valves [4]. This document details four broad categories of prosthetic valve dysfunction: structural valve dysfunction (SVD), non-structural valve dysfunction (NSVD), thrombus, and endocarditis. All forms of prosthetic valve dysfunction can cause valve stenosis and regurgitation, but the treatment for each etiology may differ. Important to note, the different etiologies of prosthetic valve dysfunction are not mutually exclusive and often co-exist [14].

SVD results from intrinsic and permanent damage to the prosthetic valve, and includes wear and tear, leaflet disruption, calcification, stent or strut fracture, or deformation [4]. The causes of SVD are thought to occur due to calcific and non-calcific mechanisms, with a combination of chemical, mechanical, immunologic contributors.

The chemicals used to fix and cross-link collagen fibers within bioprosthetic valves reduce thrombogenicity and immunogenicity, while maintaining valve integrity. These chemicals also kill valvular interstitial cells, such as fibroblasts and smooth muscles cells; consequently, and unlike native valves, there are no mechanisms by which the valves can repair themselves [15]. Additionally, prosthetic valves are less able to withstand typical mechanical forces, precipitating faster degeneration. The collagenous matrix within bioprosthetic valve leaflets is locked into a single configuration of one phase of the cardiac cycle; therefore, normal mechanical stress on inflexible leaflets promotes leaflet injury, and, subsequently, fibrosis and calcification. Lastly, chemical fixation of prosthetic valves reduces, but does not eliminate, immunogenicity. Immune-mediated mechanisms can accelerate degeneration and calcification [16, 17]. These mechanisms may explain some of the key risk factors for SVD, such as young age (mechanical stress), chronic kidney disease (premature calcification), and diabetes mellitus (inflammation) [10, 15, 17]. There is currently no medical therapy to slow the progression of SVD [17].

SVD typically occurs over many years, with a recent paper finding the 10-year re-operation rate of isolated bioprosthetic surgical aortic valve replacement (SAVR) to be ~16% [18]. The timing and indication for intervening for prosthetic valves is similar to that of native vales [1]. SVD typically occurs insidiously over many years, but it can occur rapidly and present acutely [19]. SVD is rare in mechanical valves [18, 20].

NSVD is defined as any an abnormality of the prosthesis not related to the valve, that nevertheless results in valve dysfunction [4]. Examples of NSVD include patient-prosthesis mismatch (PPM), pannus, paravalvular leaks, inappropriate positioning or sizing, or valve dysfunction related to subsequent chamber dilation after implantation.

Pannus occurs due to an abnormal immune response to the prosthesis, causing a mass of extracellular matrix and immune cells that can restrict leaflet mobility [21, 22]. The prevalence of pannus is thought to range from 0.2–4.5% and can occur in both bioprosthetic and mechanical valves [23]. Pannus and thrombus are often challenging to differentiate by echocardiography [24]. Whereas pannus typically presents longer after valve implantation and with a longer duration of symptom onset to diagnosis compared to thrombus, clinical history alone is insufficient to differentiate between these two entities. Given the very different treatment options, accurate diagnosis is key.

Valve thrombosis has a very wide spectrum of presentation, from asymptomatic and subclinical to cardiogenic shock; treatment in these scenarios differs widely and therefore accurate diagnosis is vital [24]. Thrombosis more commonly affects prosthetic valves compared to native valves and can occur in both bioprosthetic and mechanical valves, however mechanical valves are significantly more thrombogenic. In patients with mechanical valves, most cases occur due to interruption in anticoagulation [24]. When appropriately anticoagulated, the risk of valve thrombosis is similar in mechanical and bioprosthetic valves [4]. The aortic position is the least thrombogenic site for a prosthesis given the exposure to high pressure [25].

Hypoattenuating leaflet thickening (HALT) is a subclinical form of valve thrombosis that can occur in both surgical and transcatheter bioprosthetic valves [26]. When associated with reduced leaflet mobility, patients can develop hypoattenuation affecting motion (HAM) [14]. Risk factors for the development of HALT include female sex, increased age, low ejection fraction, smaller prosthesis size, under expanded transcatheter valves, and lack of anticoagulant therapy [27, 28, 29]. While the natural history and clinical significance of HALT is controversial, the diagnosis and monitoring of treatment requires a combination of echocardiography and CT [26, 29, 30].

The risk of infective endocarditis (IE) is higher among patients with prosthetic valves compared to native valves and can occur at any point in the life of the valve. Approximately 5% of patients with prosthetic valves will develop endocarditis at 10-years [31]. Pooled data from the PARTNER trials demonstrated that the majority of infections occur between 31 days and 1 year after implantation, regardless of SAVR vs transcatheter aortic valve replacement (TAVR) [32]. Prosthetic aortic valve endocarditis is associated with a very poor prognosis and definitive treatment for prosthetic valve IE almost exclusively requires surgical explantation [4, 32].

All four mechanisms can lead to both hemodynamically significant stenosis and regurgitation or a combination of both.

## 4. Transthoracic Echocardiography

TTE is the cornerstone imaging modality for the assessment of prosthetic valves and should be the initial test of choice when any form of prosthetic valve dysfunction is suspected [4].

### 4.1 Baseline Echocardiography and Monitoring in Asymptomatic Patients

Determining the extent and severity of prosthetic valve dysfunction must be done in the context of the baseline characteristics of a prosthetic valve. Defining the initial gradients of a valve is therefore vital to monitoring the ongoing function of the valve. The ideal period to perform the baseline exam is 1 to 3 months after the procedure when the chest wall has healed, and therefore all the proper acoustic windows can be more readily accessed, and when post-operative anemia has normalized, preventing falsely elevated gradients in the setting of a high-flow state [1]. Several web-based applications exist that describe the expected gradients based on manufacturer recommendation. However, the “normal” baseline gradient will vary for each patient depending on their type of prosthesis, size of prosthesis, and flow characteristics [4].

The frequency of routine TTE evaluation in asymptomatic patients is controversial. The most recent American College of Cardiology/American Heart Association guidelines do not recommend additional TTE in asymptomatic patients with normally functioning mechanical valves on baseline imaging. Surgical bioprosthetic valves should be imaged 5 and 10 years after surgery, and then annually thereafter. Transcatheter valves should be imaged annually [1]. Alternatively, the most recent European Society of Cardiology guidelines recommend echocardiography at 1 year and then annually for all bioprosthetic valves [2].

### 4.2 Prosthetic Aortic Stenosis and its Mimickers

A thorough interrogation of prosthetic aortic valves involves direct visualization of leaflet morphology and mobility along with color and spectral Doppler assessment. Similar to the interrogation of native valves, peak velocity, peak and mean gradients, dimensionless index, effective orifice area (EOA), and jet contour are important measures of valve function. For assessment of prosthetic aortic valves, a greater emphasis is placed on acceleration time (AT) and ejection time (ET), as these measures can be helpful to differentiate prosthetic aortic stenosis from mimickers. AT is the time interval between the beginning of systolic flow and its peak velocity on continuous-wave Doppler. ET is the time of onset from valve opening to valve closing [33, 34]. A valve without significant stenosis should have an AT <100 ms and an AT/ET ratio of <0.37 [4].

The hemodynamic criteria for valve deterioration have been defined and classified into two groups: possible and significant valve degeneration (Table 2) [4]. It is important when there is concern for prosthetic aortic valve stenosis to rule out mimickers that can result in elevated gradients, such as aortic regurgitation, high-flow states, PPM, and pressure-recovery phenomenon.

**Table 2.** **Hemodynamic criteria for valve deterioration**.

	Possible structural valve degeneration	Significant structural valve degeneration
Prosthetic valve stenosis	Increase in mean transvalvular gradient ≥10 mmHg resulting in a mean gradient ≥20 mmHg	Increase in mean transvalvular gradient ≥20 mmHg resulting in a mean gradient ≥30 mmHg
	— AND —	— AND —
	Decrease in effective orifice area ≥0.3 cm^2^ or ≥25%	Decrease in effective orifice area ≥0.6 cm^2^ or ≥50%
	— OR—	— OR—
	Decrease in dimensionless index ≥0.1 or ≥20%	Decrease in dimensionless index ≥0.2 or ≥40%
Prosthetic valve regurgitation	New occurrence or increase of at least one grade of intraprosthetic AR resulting in moderate or greater AR	New occurrence or increase of at least two grades of intraprosthetic AR resulting in moderate or greater to severe AR

AR, aortic valve regurgitation.

**Fig. 1.**
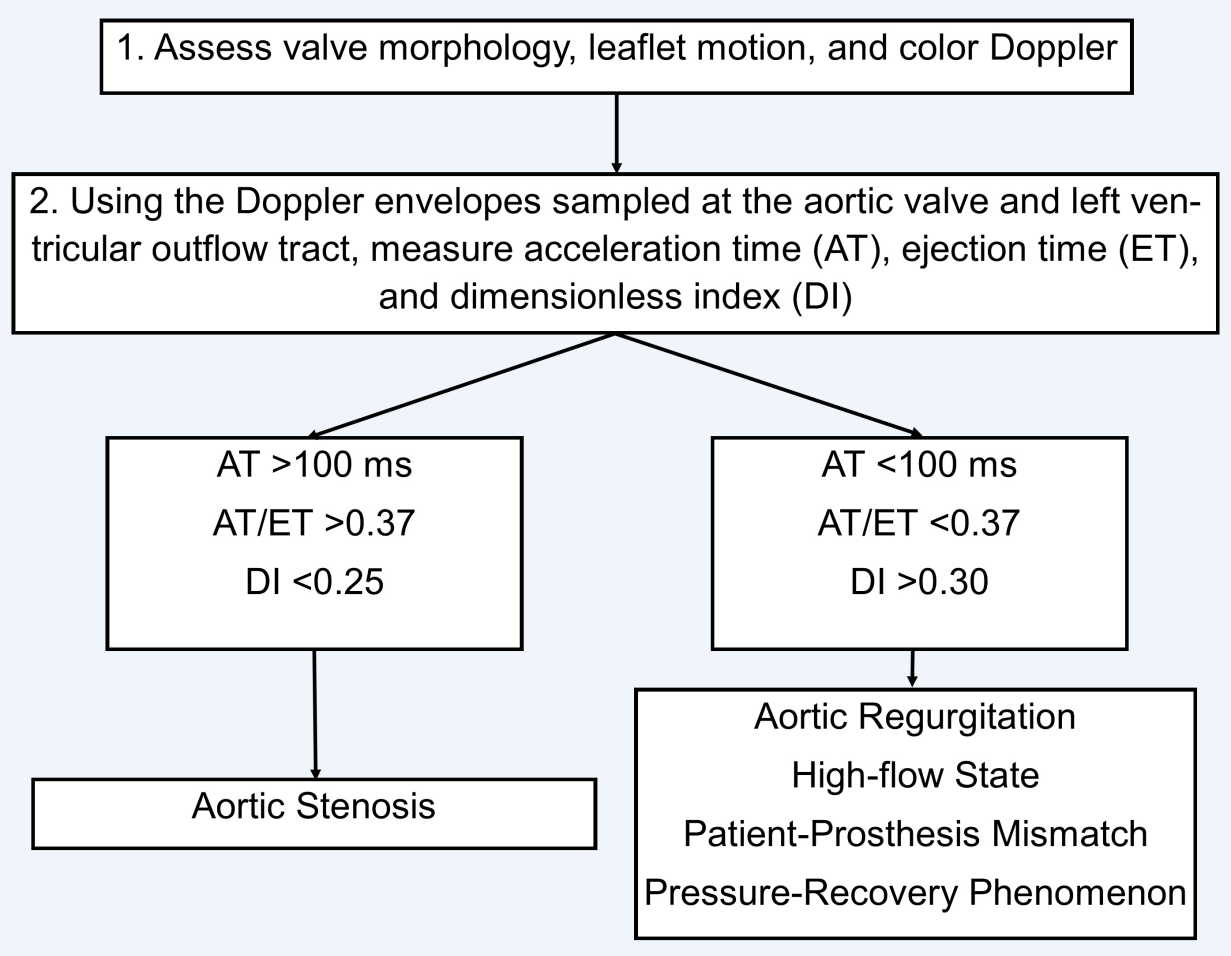
**Algorithm for differentiating prosthetic valve aortic stenosis versus mimickers in the setting of elevated gradients**.

**Fig. 2.**
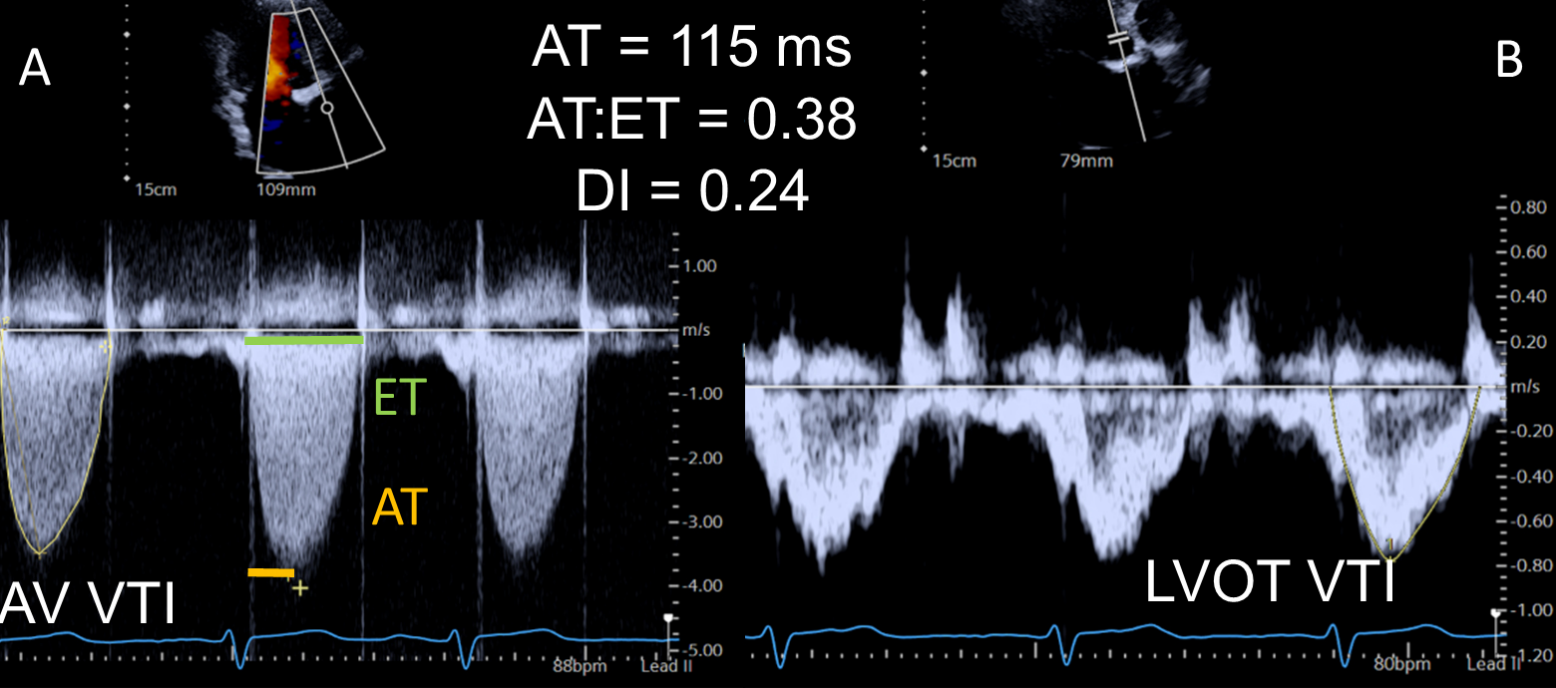
**Prosthetic aortic stenosis on transthoracic echocardiography**. Patient with #23 Carpentier Edwards bioprosthetic aortic valve with severe stenosis (peak/mean gradient 50/31 mmHg). (A) Doppler profile shows late-peaking signal (acceleration time (AT) 115 ms) with abnormal acceleration time to ejection time (ET) ratio (0.38). The aortic valve time-velocity integral (AV VTI) was 82 cm and the left ventricular outflow tract time-velocity integral (LVOT VTI) 20 cm (B), resulting in a dimensionless index (DI) of 0.24. These findings are concerning for true aortic stenosis.

When trying to ascertain whether elevated gradients are due to stenosis, the American Society of Echocardiography recommends initially evaluating jet contour, AT, ET, and dimensionless index (DI). Elevated gradients with an early peak (AT <100 ms, AT/ET <0.37) and normal DI (>0.3) are more consistent with a high flow state, patient-prosthesis mismatch, or pressure-recovery phenomenon, whereas elevated gradients with a late peak (AT >100 ms, AT/ET >0.37) and low DI (<0.25) are more consistent with true stenosis (Figs. 1,2). When there are discrepancies between AT, AT/ET, and DI, it is important to consider technical errors, such as improper sampling of the left ventricular outflow tract (LVOT) or poor alignment of the Doppler signal [4].

Differentiating PPM from stenosis, high-flow states and pressure-recovery phenomenon is important, as several studies have shown worse all-cause mortality and rehospitalization for patients with severe PPM [35, 36]. PPM occurs when the prosthesis is too small for the body size and is defined as an indexed EOA of <0.85 cm^2^/m^2^, with severe PPM as <0.65 cm^2^/m^2^. In obese patients, these cutoffs may lead to overdiagnosis of PPM and therefore the guidelines recommended using cutoffs of 0.7 cm^2^/m^2^ and 0.55 cm^2^/m^2^ for patients with body mass index >30 kg/m^2^ [4, 37]. Valve manufacturers report an expected EOA, however EOA derived from the continuity equation is the methodology that has been consistently linked to poor outcomes [4].

When distinguishing true stenosis from PPM, having access to the baseline gradients is important; in cases of PPM, as opposed to prosthetic aortic stenosis, elevated gradients should be present immediately after placement of the valve. EOA indexed to body surface area should remain largely stable in the presence of PPM, barring large shifts in body habitus [4]. Aortic root enlargement, allowing for placement of a larger prosthesis, is an option to both prevent PPM and treat severe, symptomatic PPM [38].

Pressure-recovery phenomenon can also cause high gradients by echocardiographic and can be mistaken for aortic stenosis. Significant pressure-recovery phenomenon is typically seen in patients with smaller aortas (<3 cm) and bileaflet mechanical valves. Bileaflet mechanical valves have three orifices, two lateral and one central. Because the central orifice is smaller than the lateral ones, blood flows at a higher velocity through it, resulting in a greater drop in pressure, and subsequently a higher-pressure gradient. This pressure drop “recovers” when flow from the lateral orifices joins with the flow from the central orifice. Doppler echocardiography is unable to distinguish the velocities between the central and lateral orifices, leading to an overestimation of the gradient. Confirmation of elevated gradients due to pressure-recovery phenomenon can be performed with direct pressure measurements via invasive catheterization [39].

Elevated gradients in the absence of stenosis, PPM, pressure-recovery phenomenon, or aortic regurgitation likely reflects a high-flow state, such as anemia, sepsis, or hyperthyroidism [4].

### 4.3 Prosthetic Aortic Regurgitation

Similar to prosthetic aortic stenosis, the hemodynamic criteria for valve degeneration for aortic regurgitation have been defined (Table 2) [4]. When evaluating prosthetic aortic valve regurgitation, distinguishing valvular vs paravalvular regurgitation is key, as the therapy for these two valvular pathologies can be different. Depending on the etiology of valvular regurgitation, patients may benefit from either SAVR or valve-in-valve TAVR However, if the pathology is paravalvular, paravalvular leak closure may be a safe and effective procedure [40, 41].

**Fig. 3.**
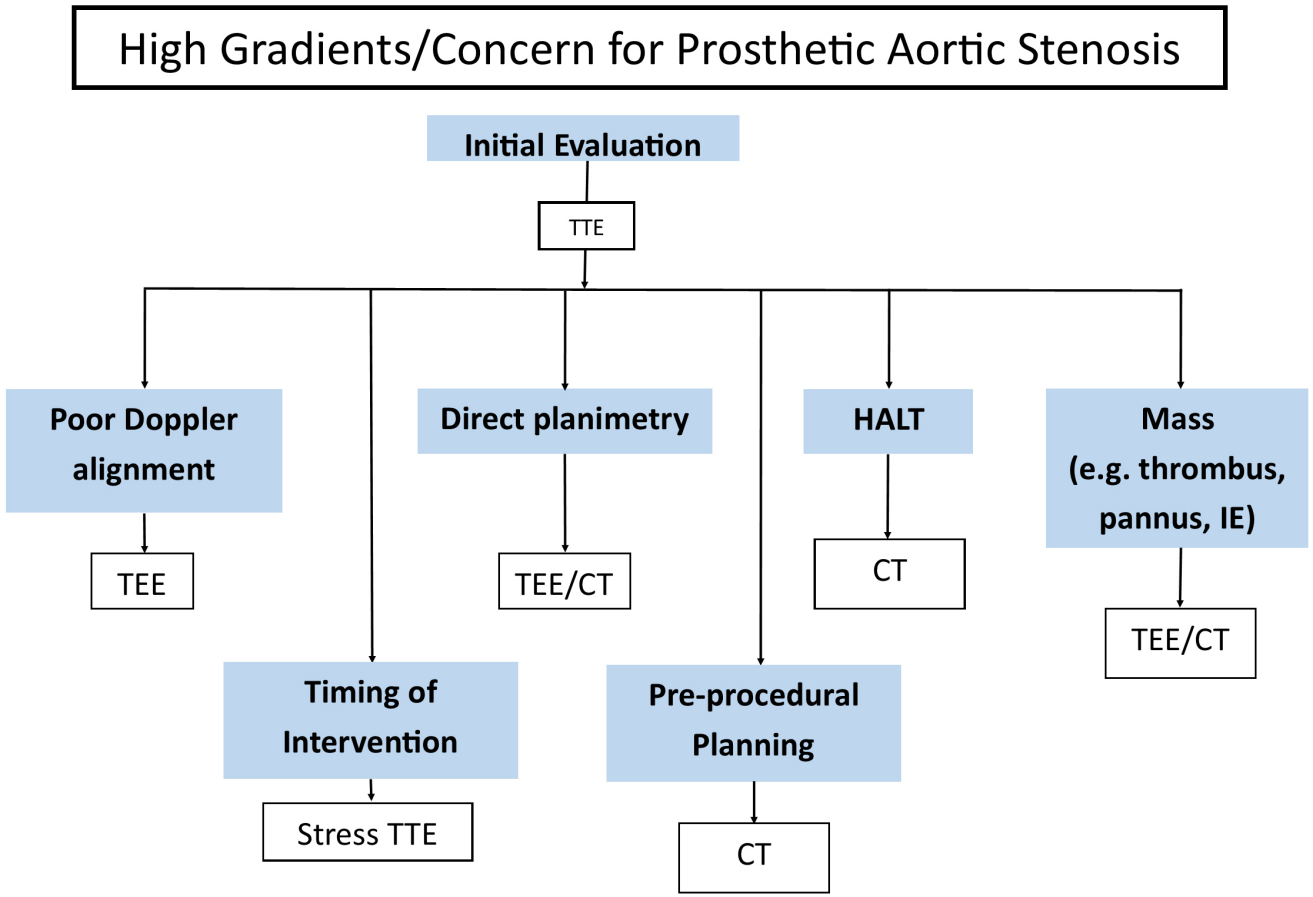
**Common clinical indications for multimodality imaging for the assessment of prosthetic aortic stenosis**. TEE, transesophageal echocardiography; TTE, transthoracic echocardiography; CT, computed tomography; HALT, hypoattenuating leaflet thickening; IE, infective endocarditis.

Among surgical valves, paravalvular leak primarily occurs via suture dehiscence, which can occur from a variety of etiologies, including endocarditis, poor tissue integrity, or poor surgical technique [42]. For transcatheter valves, inaccurate prosthetic sizing, asymmetric valvular calcium, and left ventricular outflow angle can all contribute to incomplete apposition of the valve in the annulus [43]. The rate of moderate-severe paravalvular leak is low amongst both balloon expandable (0.5–3.7%) and self-expandable valves (3.4–5.3%). The prevalence of moderate-severe PVL was also low (<1%) among surgical valves in the various TAVR trials [44, 45, 46, 47]. A scheme for grading paravalvular leak has been proposed and was incorporated into the later TAVR trials. This scheme recommends measuring the circumferential extent of the leak on color Doppler, with 20–30% representing moderate AR and >30% representing severe AR [48]. Fig. 3 shows common indications for multimodality imaging when there is concern for prosthetic aortic valve stenosis.

### 4.4 Stress Echocardiography

Stress echocardiography has a key diagnostic role in the evaluation of many types of native valve dysfunction. In patients for whom symptoms are unclear, stress echocardiography can objectively measure functional capacity, link patient’s symptoms more clearly to exertion, and assess left ventricular response to exercise [49]. In particular, the role for exercise stress echocardiography in patients with asymptomatic severe AS is well-defined, with a normal exercise stress echocardiogram associated with a very low risk of cardiac death at 1 year [50]. The role of stress echocardiography is less well-defined in aortic regurgitation, but can still be used to objectively assess functional capacity and symptoms and to determine contractile reserve [51]. Given the shorter lifespans of prosthetic aortic valves, along with the higher risks associated with repeat interventions, stress echocardiography is an excellent tool to help determine the optimal time to intervene on prosthetic aortic valve dysfunction when symptoms are equivocal [52].

## 5. Transesophageal Echocardiography

There are several clinical scenarios for which TEE can be a useful adjunct to TTE to aid in the diagnosis of prosthetic valve pathology. The superior spatial resolution of TEE, along with its proximity to the heart and its posterior imaging position, can better characterize many prosthetic aortic valve pathologies. However, many of the limitations of TTE in this population also apply to TEE [4].

### 5.1 Prosthetic Valve Assessment

TEE can be useful in the assessment of elevated prosthetic aortic valve gradients when transthoracic imaging is poor. Leaflet or occluder mobility can frequently be visualized in the mid-esophageal views, but, like parasternal long axis imaging on TTE, these structures can be obscured by acoustic shadowing from the prosthesis. Transgastric imaging can often provide excellent alignment for Doppler assessment of both the valve and the left ventricular outflow tract, while also allowing for visualization of leaflet mobility. In the presence of elevated gradients, but with normal leaflet mobility, gastric images may help clinicians diagnose PPM, high output, or, most importantly, significant AR [53].

Defining the severity and mechanism of aortic regurgitation is a key indication for TEE for prosthetic aortic valves. Obtaining diagnostic imaging of eccentric aortic regurgitation jets can be challenging on TTE. Accurately assessing severity of aortic regurgitation requires optimal Doppler alignment and visualization of the origin of the jet, allowing for measurement of vena contracta size and proximal isovolumetric surface area; neither of these metrics are easily measured on transthoracic imaging in the setting of a prosthesis. The combination of mid-esophageal and gastric views on TEE can often provide acoustic windows with better visualization of jet origin and better Doppler alignment [4].

**Fig. 4.**
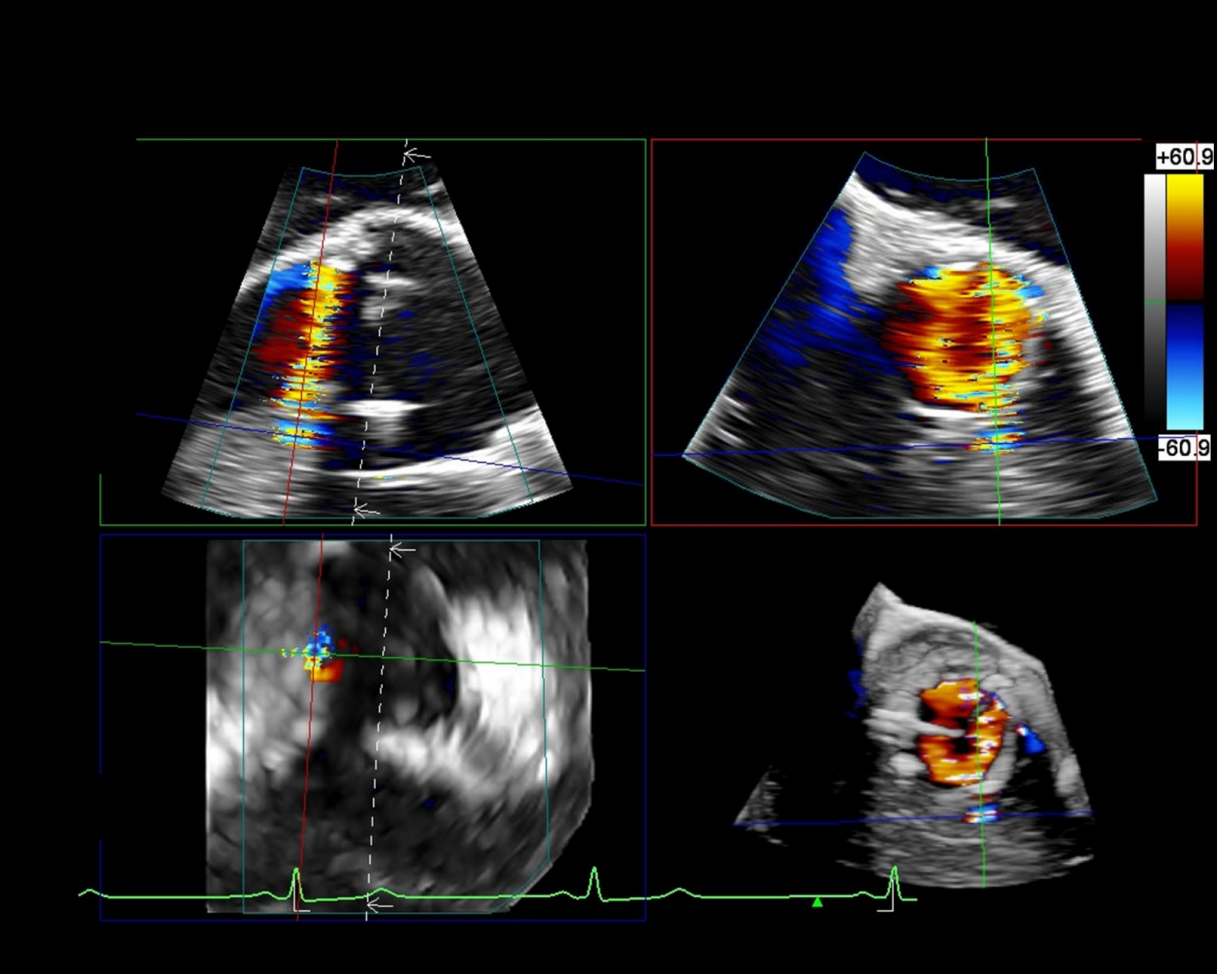
**Transesophageal echo with paravalvular leak**. Patient with #27 Inspiris surgical bioprosthetic aortic valve who presents with moderate aortic regurgitation of unclear mechanism on transthoracic echocardiography. Transesophageal echocardiography, using multiplanar reconstruction, demonstrates a paravalvular leak at the 5 o’clock position.

**Fig. 5.**
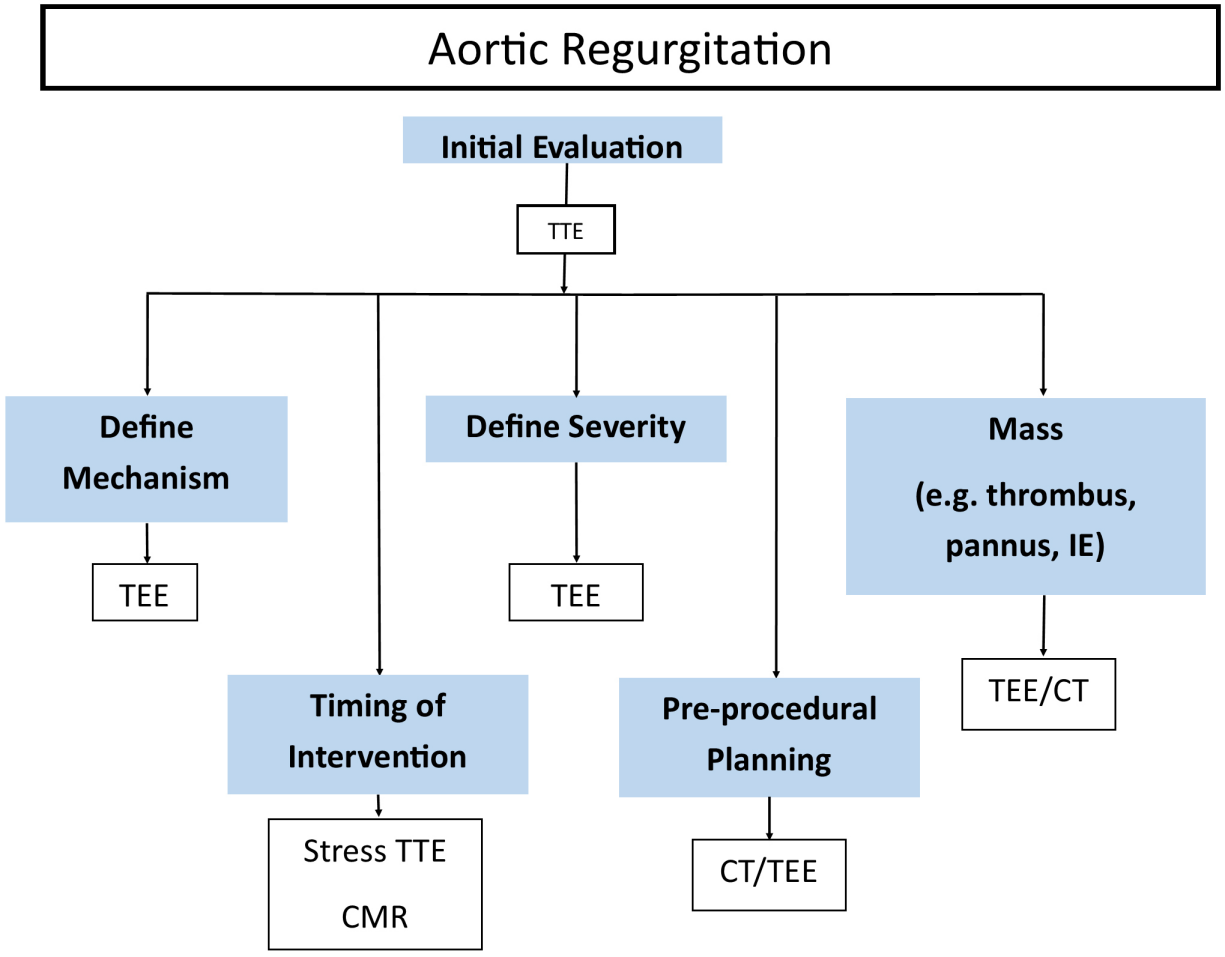
**Common clinical indications for multimodality imaging for the assessment of prosthetic aortic regurgitation**. TEE, transesophageal echocardiography; TTE, transthoracic echocardiography; CT, computed tomography; CMR, magnetic resonance imaging; IE, infective endocarditis.

The most important indication for TEE in the assessment of prosthetic aortic valve regurgitation is the determination of whether the regurgitation is valvular or paravalvular. One comparative study found TEE to be superior to TTE for the identification of AR mechanism, with correct characterization in 88% of cases compared to 54% by TTE [54]. TEE can be helpful in determining the number, location, size, and severity of paravalvular leaks, particularly with the addition of 3D TEE. 3D TEE with multiplanar construction allows for more precise description and localization of pathology, which can improve pre-procedural planning and aid in selection of paravalvular closure device sizing (Fig. 4) [55]. Percutaneous closure of paravalvular leaks is a safe and effective procedure for the treatment of both hemodynamically significant leaks and hemolysis [41, 55, 56]. Fig. 5 shows common clinical indications for multimodality imaging in the setting of prosthetic aortic regurgitation.

### 5.2 Endocarditis

**Fig. 6.**
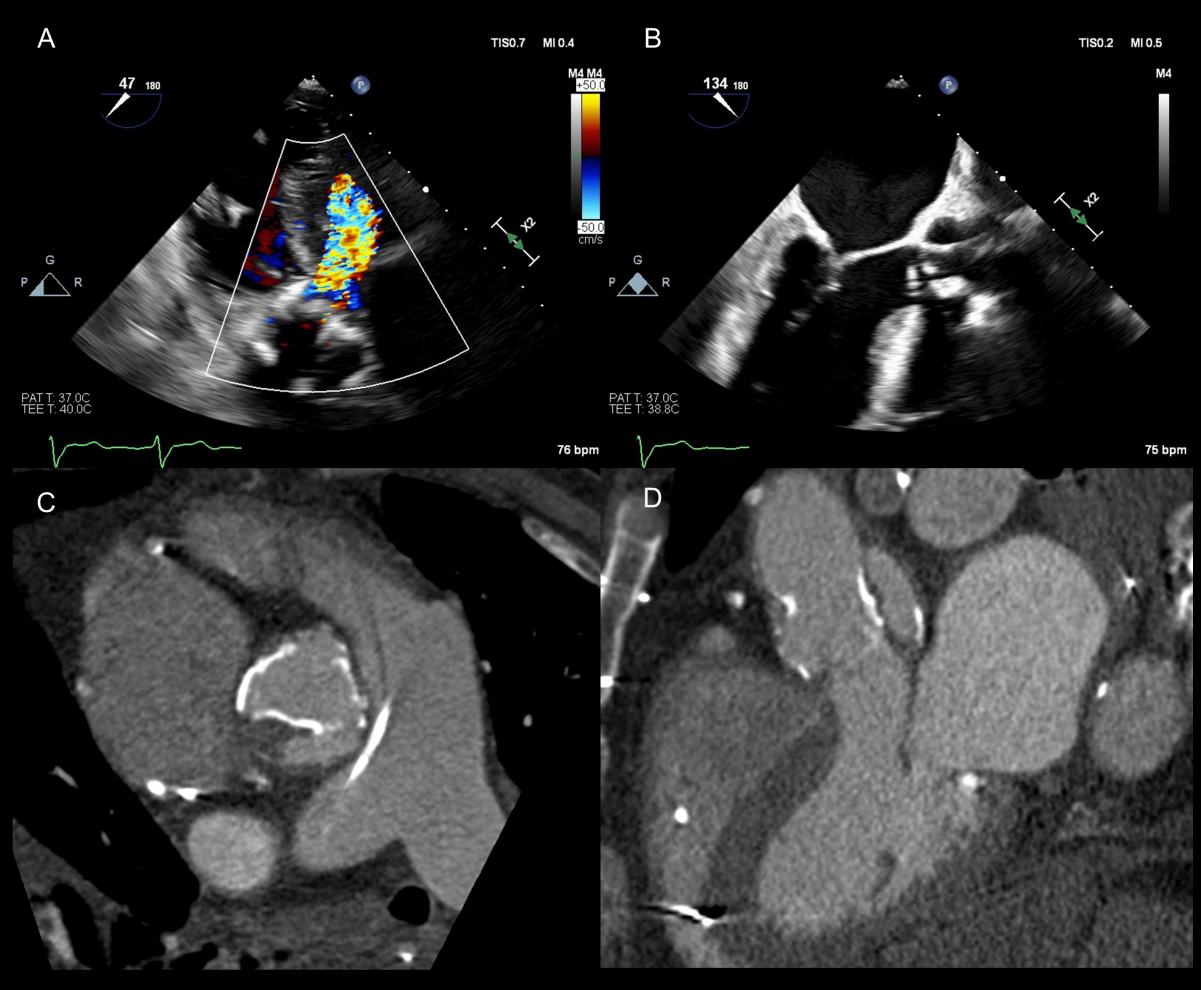
**Endocarditis with paravalvular abscess and pseudoaneurysm**. Patient with severe aortic regurgitation due to endocarditis (A). There is evidence of thickening around the aortic root on transesophageal echocardiography (TEE) concerning for root abscess (B). Cardiac computed tomography (CT) confirms aortic root abscess and partially thrombosed pseudoaneurysm (C,D). CT better demonstrates the extent of annular complication than TEE. PAT, patient temperature.

Prosthetic valve endocarditis is common, with an incidence of 0.3–5.9 cases per 100 person-years [57, 58]. Mortality for prosthetic valve endocarditis is significantly higher than native valve endocarditis, with high in-hospital (14–22%) and 1-year mortality (40%) [59]. Among those with TAVR, up until recently reserved for higher risk patients, the mortality rates are even worse, with in-hospital mortality ranging from 16–64% [60, 61, 62, 63]. Key to understanding the worse outcomes with prosthetic valve endocarditis is an appreciation of complicated or invasive disease. Patients with prosthetic valves are significantly more likely to develop invasive disease such as dehiscence, abscess, pseudoaneurysm, or fistula (Fig. 6). Patients with invasive disease have significantly worse outcomes [64, 65, 66].

TTE has poor sensitivity in detecting native valve endocarditis; given the presence of acoustic shadowing, TTE has even worse performance when assessing prosthetic valves for infection. Studies have found the sensitivity of TTE for prosthetic valve endocarditis to range from 17–36% [67, 68]. TEE provides significantly better sensitivity for the detection of vegetations, ranging from 82 to 96% [54]. There has been a trend towards even greater diagnostic accuracy for TEE with the greater utilization of 3D-echocardiography [67, 68, 69]. 3D TEE allows for better visualization of vegetation size and location, along with destructive changes, paravalvular leaks, and valve dehiscence [70, 71].

Another significant advantage of TEE over TTE is the detection of annular complications. Abscesses are more common with prosthetic aortic valves and frequently involve the aorto-mitral curtain. Abscesses typically present as a hypoechoic thickening around the aortic root without associated color flow; color flow into a periannular lesion may represent pseudoaneurysm or fistula [72]. Low sensitivity for the detection of aortic root abscess, ranging from 18 to 28% [54, 73]. Studies have found the sensitivity for TEE to range from 70–88% [73, 74, 75]. For these reasons, TEE is a class I indication in the most recent European Society of Cardiology (ESC) guidelines in all patients with prosthetic heart valves with a clinical suspicion for IE [76].

A repeat TEE within 5–7 days in patients with an initial negative exam for which the clinical suspicion of endocarditis remains is an additional class I indication [76]. This recommendation is based on data in all-comers, not just prosthetic valve endocarditis, that have shown a significant minority of patients with a negative TEE can have a clinical change after a short interval. Prior studies found between 5–17% of patients with an initial negative TEE, but continued clinical suspicion for endocarditis, were subsequently found to have endocarditis on a later TEE [77, 78, 79]. Furthermore, repeat TEE can often have a clinical impact on antimicrobial therapy and decisions to pursue surgery [77].

## 6. Cardiac Computed Tomography

With significant improvements in the temporal resolution and gating of CT over the last few decades, cardiac CT has become a fundamental imaging modality in the assessment of cardiovascular disease, particularly the assessment of prosthetic valves [80].

Cardiac CT is typically performed on multi-detector platforms and with acquisition precisely timed with the patient’s electrocardiogram (ECG), known as gating. Three different acquisition techniques are used in cardiac CT: prospective/axial sequential, high-pitch spiral/“flash”, and retrospective/spiral helical. Prospective ECG-gating acquires images over multiple beats at a specific part of the R-R interval; this modality significantly reduces the amount of radiation but is more susceptible to arrythmia and patient motion. With high-pitch spiral acquisition, the patient is moved rapidly through the scanner (fast pitch), allowing for acquisition of the images in one cardiac beat. This method significantly reduces radiation exposure but is also susceptible to motion artifact; additionally, this type of acquisition is not gated and therefore may fall during any part of systole or diastole. In retrospective ECG-gating, data is acquired continuously as the patient moves through the scanner; while this type of gating increases radiation, it allows for assessment of the heart throughout the cardiac cycle. 4-dimensional (4D) CT with iodinated contrast uses retrospective ECG-gating without radiation attenuation and is the acquisition technique of choice when assessing the morphology and function of prosthetic valves [81].

### 6.1 Restricted Leaflet Motion

Due to acoustic shadowing, particularly with mechanical valves, echocardiography is often limited in direct visualization of valve leaflets or occluders; conversely, CT has excellent spatial and temporal resolution for the assessment of leaflet and occluder mobility [4, 14]. CT can be particularly helpful in the setting of elevated gradients where the diagnosis of valve stenosis cannot clearly be made by echocardiography. With 4D CT with iodinated contrast, leaflet thickness and mobility can be clearly seen. This can aid in the differentiation of stenosis versus mimickers, such as prosthesis-patient mismatch, pressure-recovery phenomenon, or high-flow states, where leaflet mobility is normal.

It is important to note that echocardiography and CT are assessing two different measures of valve area: EOA and geometric orifice area (GOA). EOA, as measured by echocardiography, uses the continuity equation; GOA is a direct measurement of area by planimetry [82]. Among native valves, the EOA is typically smaller than the geometric orifice area by an average of 0.1–0.2 cm^2^, although the degree to which this is true is patient-specific and is affected by leaflet calcification, leaflet shape, and aorta size [82, 83]. An echocardiography/CT comparison study in TAVR valves found a similar relationship between EOA and GOA in patients undergoing evaluation for patient-prosthesis mismatch; this explains why the prevalence of patient-prosthesis mismatch was lower when measured by CT than when measured by TTE. In this study, CT-defined, but not TTE-defined, patient prosthesis mismatch was associated with mortality [84].

The severity of aortic leaflet calcification by Agaston units has been well-studied to correlate with severity of native valve aortic stenosis, and diagnostic thresholds have been defined for men and women [1, 85, 86]. Bioprosthetic valves should not have calcification and any calcification signals degeneration of the leaflets. However, specific thresholds to define severity of prosthetic valve dysfunction by degree of calcification have not been studied.

### 6.2 Pannus Versus Thrombus

Differentiation of pannus and thrombosis is extremely challenging by echocardiography; clinical history, rather than imaging, has traditionally been more important for the diagnosis of these entities. Thrombosis traditionally occurs closer to the date of implant and presents more acutely, whereas pannus typically occurs later in the valve’s lifetime and the progression of dysfunction is more insidious [87]. There are certain imaging characteristics more indicative of one etiology vs the other. Almost all cases of thrombosis involve abnormalities in leaflet motion versus only 60% in cases of pannus. Pannus tends to be circumferential and grow inward from the valve annulus, whereas thrombus can be bulkier and more irregular. Nevertheless, these findings are not specific enough to warrant a high degree of diagnostic confidence, particularly since pannus and thrombus can often co-exist [4, 24]. Diagnosis is of key importance; while both pathologies are often treated with surgical explantation and valve replacement, some patients with thrombus can be treated with anticoagulation or fibrinolysis [88, 89]. Fibrinolysis comes with significant risks and therefore accurate diagnosis is paramount [89].

Cardiac CT has emerged as a valuable tool for the differentiation of pannus and thrombus. Previous studies had noted higher Hounsfield units (HU) for pannus compared to thrombus [23]. This prompted a prospective, observational trial by Gündüz *et al*. [90] evaluating CT in patients being treated for mechanical valve thrombosis. The authors found two HU thresholds that can aid with diagnosis and predict response to fibrinolysis. Valve masses with <90 HU had a 100% response to fibrinolytics compared to only 42.1% of those with HU between 90 and 145. HU >145 was associated with a significantly lower rate of complete/partial lysis (33%) and complete lysis (6.3%). Of the patients with valve mass of >140 HU, 82% had pannus alone and an additional 12% had pannus and thrombus. This study suggests that low HU (<90) and high HU (>145) can accurately diagnose thrombus and pannus, respectively [90].

### 6.3 Hypoattenuating Leaflet Thickening

**Fig. 7.**
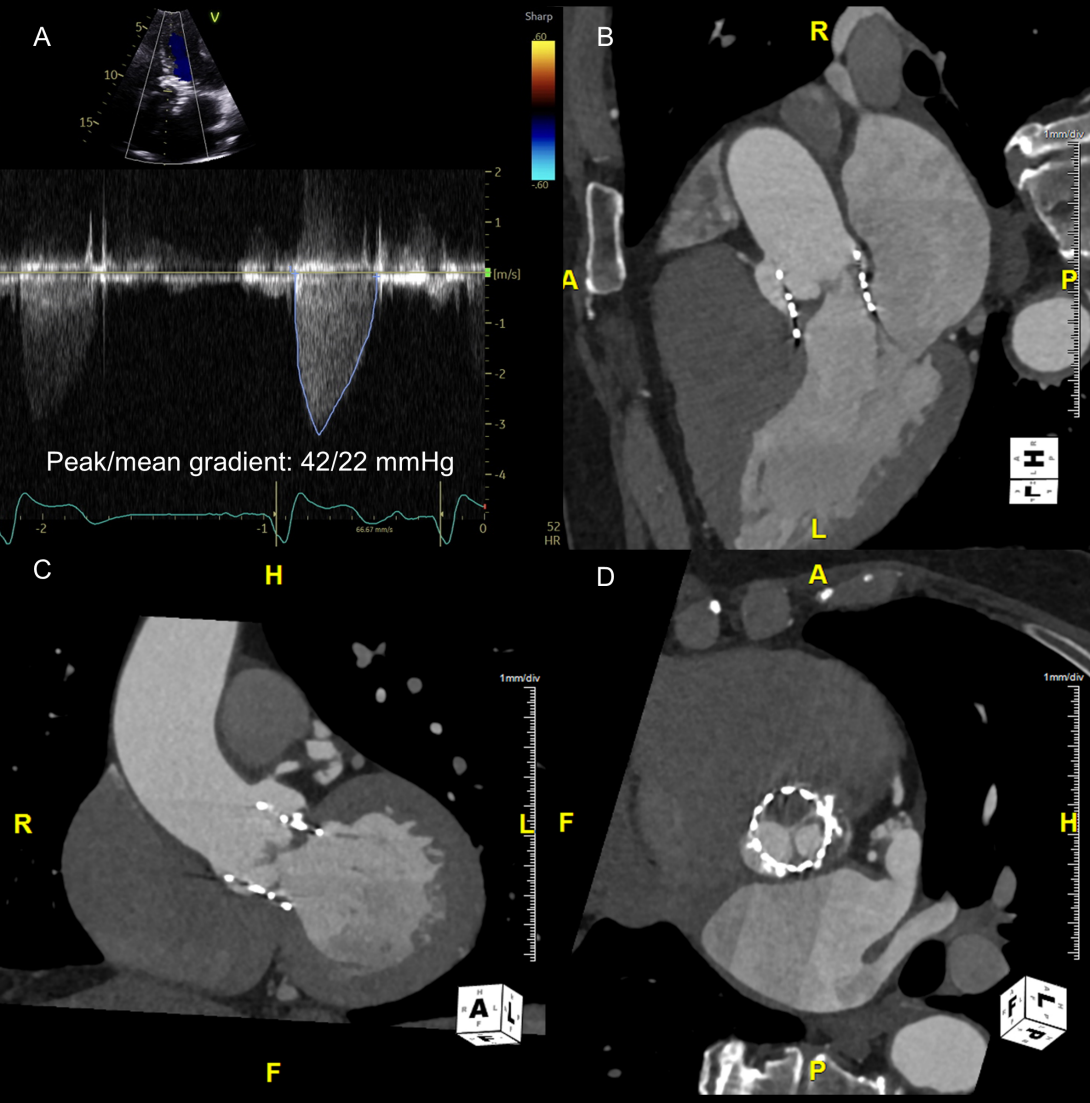
**Hypoattenuating leaflet thickening with restricted leaflet motion**. Patient with a 29 mm Edwards Sapien S3 transcatheter aortic valve developed increased gradients from baseline (A). Cardiac 4D computed tomography shows severe hypoattenuating leaflet thickening and restriction of the right coronary cusp equivalent leaflet and mild hypoattenuating leaflet thickening of the left coronary cusp equivalent leaflet (B–D). 4D, 4-dimensional.

Cardiac CT is the test of choice for the evaluation of HALT. Both the Society of Cardiovascular Computed Tomography and the Valve Academic Research Consortium have similar recommendations on the interpretation and reporting of HALT [91, 92]. When evaluating for the presence of HALT on a 4D CT with contrast, specific mention should be made for percentage of leaflet involvement, the number of leaflets involved, and whether there is restricted leaflet motion, also known as HAM. The percentage of leaflet involvement is usually classified as <25%, 25–50%, 50–75%, and >75% (Fig. 7).

The clinical and therapeutic implications of HALT are controversial. The most robust data on HALT derive from the prospective, randomized trials of transcatheter and surgical aortic valves. Whereas prior registries suggested higher rates of HALT with TAVR, analysis of more recent, prospective trials found similar rates of HALT between surgical and transcatheter valves, regardless of TAVR platform [26, 27, 28].

Many studies have linked the presence of HALT with worse outcomes, including all-cause mortality, cardiovascular-mortality, and cerebrovascular events. However, the mechanisms linking HALT to these poor outcomes are unclear. The hemodynamic effects of HALT on the valves are also not clear. Some studies show elevated gradients with HALT, some show elevated gradients with only severe leaflet involvement, and some show no associated between HALT and increased gradients [26, 27, 29, 30].

The natural history of HALT was characterized in a sub-study of the Placement of Aortic Transcatheter Valves (PARTNER) low-risk study. Patients underwent CT for the evaluation of HALT at 30 days and 1 year. The prevalence of HALT was higher at 1 year (30%) than at 30-days. Notably, 54% of the patients with HALT at 30-days had spontaneous resolution; conversely, 21% of patients who had no HALT at 30 days developed HALT at 1 year [26]. The long term effects of HALT on the durability of valves is unknown.

Due to the mixed data on the significance of HALT, along with the gaps in knowledge on the long-term effects of HALT, the Society of Cardiovascular Computed Tomography, as part of a 2019 expert consensus, recommended against routine CT imaging following TAVR. CT can be considered when there is clinical concern for valve dysfunction by echocardiography, such an increase in gradients or decrease in leaflet mobility [91].

### 6.4 Valve-in-Valve Interventions

Cardiac CT is required for pre-procedural planning of valve-in-valve TAVR. Coronary artery obstruction can be a devastating consequence of TAVR. The risk of coronary obstruction is significantly higher in valve-in-valve TAVR compared to native valve TAVR [93]. During valve-in-valve TAVR, the new valve displaces the prior bioprosthetic leaflets into an open position against the frame of the original valve, causing a “covered cylinder” [94]. Contrary to native valve TAVR, coronary height, sinotubular junction height, and sinus of Valsalva width are inadequate to predict the risk of coronary obstruction.

**Fig. 8.**
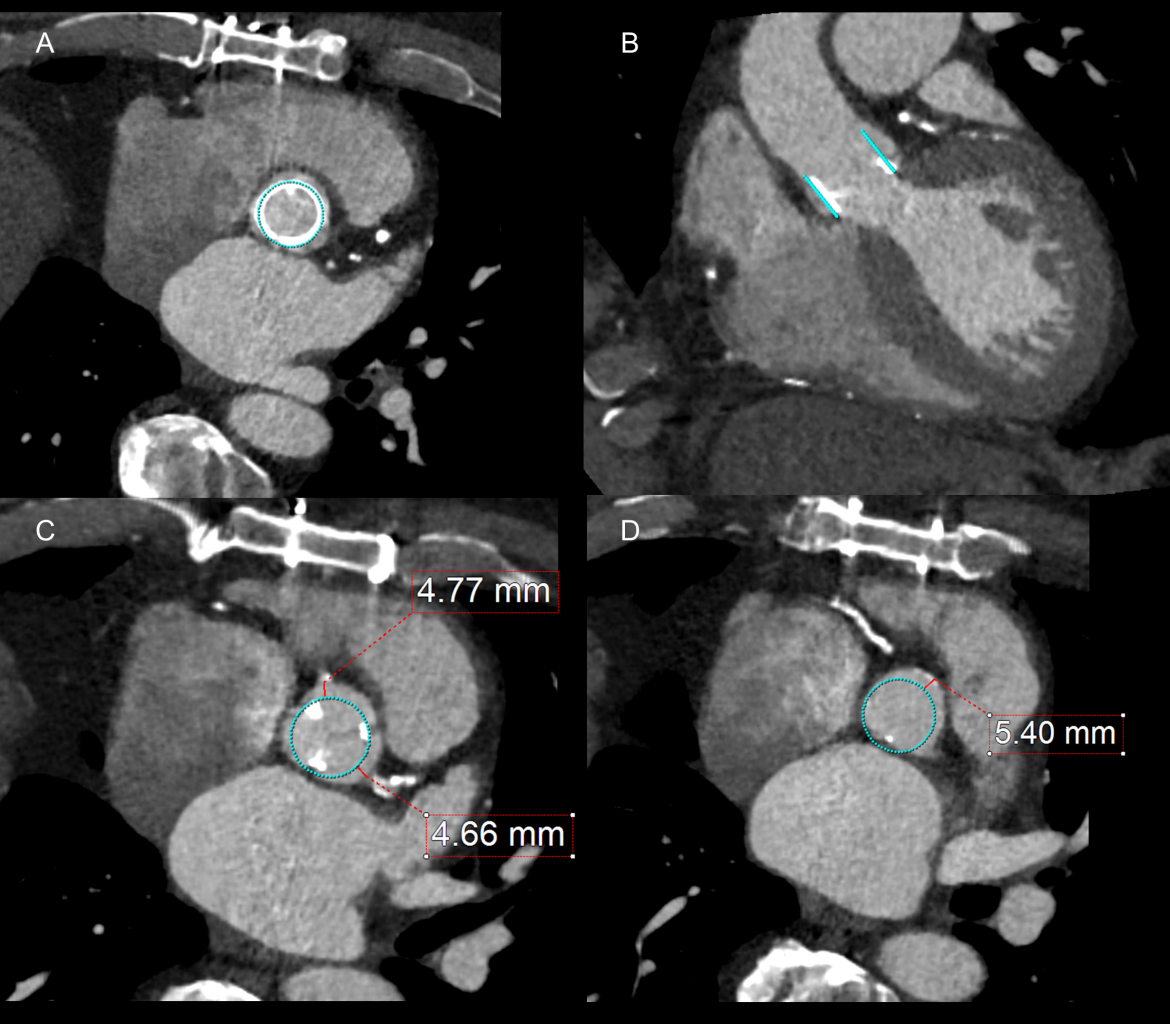
**Planning valve-in-valve transcatheter aortic valve replacement**. Patient presented with severe aortic stenosis in a #25 Carpentier Edwards bioprosthetic aortic valve and underwent valve-in-valve transcatheter aortic valve replacement. (A,B) demonstrate embedded virtual prosthesis. The virtual prosthesis is used to measure virtual transcatheter heart valve to coronary distance (VTC) (C) and virtual transcatheter heart valve to sinotubular junction distance (VTSTJ) (D). A VTC >4 mm and a VTSTJ >2 mm is considered low risk of coronary obstruction.

Several indices have been proposed to predict the risk of coronary obstruction in valve-in-valve TAVR: virtual transcatheter heart valve to coronary distance (VTC) and the virtual transcatheter heart valve to sinotubular junction distance (VTSTJ). Both of these metrics involve using current CT software to virtually embed the proposed TAVR valve into a 4D CT. VTC is the distance between the virtual valve and the coronary ostia and VTSTJ the distance from the virtual valve to the sinotubular junction (Fig. 8) [95].

The VTC was validated in a multicenter study that found a VTC <4 mm to be predict coronary obstruction [93]. Similarly, a VTSTJ of <2 mm is also thought to significantly increase risk of coronary obstruction [96]. These cutoffs were used in a prospective trial evaluating the BASILICA device, a transcatheter electrosurgical device to lacerate aortic leaflets prior to TAVR and therefore prevent coronary obstruction. In this trial, there were no cases of coronary obstruction in valve-in-valve TAVR with VTC <4 mm with the use of the BASILICA device. VTC and VTSTJ have been combined to further define the risk of coronary obstruction, classify patients into low-, intermediate-, and high-risk categories for coronary obstruction, and guide the use of leaflet modification devices [95].

In the evaluation for valve-in-valve TAVR, CT can also assess the degree of peripheral arterial disease, the extent of coronary artery calcifications, the quantification of chamber size and function, and the identification of non-cardiac pathologies [91]. CT is essential for determining the access site for TAVR [97]. A transfemoral approach is the preferred for the strategy of all TAVR valves, however, in patients with prohibitive vascular anatomy, many alternative-access sites have been described, including transapical, transaortic, transcaval, transcarotid, subclavian/axillary, and suprasternal [98]. Recent data suggests percutaneous treatment of the underlying peripheral arterial disease at the time of the TAVR may be preferred over alternative-access, however, this is not an option for all patients [99]. The optimal alternative access site is center-specific as it is primarily determined by local-expertise and experience [100].

### 6.5 Paravalvular Leak

Paravalvular leaks can frequently be identified on cardiac CT. A study comparing CT to echocardiography performed within 7 days for the assessment of paravalvular leak found CT to highly sensitive; CT also had comparable size measurements to echocardiography [101]. During interpretation of paravalvular leaks by CT, it is important to properly window the images in order to reduce beam hardening artifact. This artifact, along with the presence of suture material, can frequently cause misinterpretation on paravalvular leaks [102].

### 6.6 Endocarditis

CT has become an integral adjunct to echocardiography in the assessment of endocarditis, particularly prosthetic valve endocarditis, due to its superiority in imaging periannular complications. 4D CT can often identify vegetations, valve dehiscence, pseudoaneurysms, abscess, leaflet perforations, fistulas, mycotic aneurysms, and embolic phenomena to other organs.

Compared to 4D CT, TEE has superior sensitivity (89 vs 78%), albeit with lower specificity (74 vs 94%), for the detection of vegetations, particularly vegetations smaller than 5 mm. TEE is also more sensitive for the detection of leaflet perforation (79 vs 48%) [58, 74].

Conversely, CT is the superior imaging modality for the detection of periannular complications, such as paravalvular abscess or pseudoaneurysm (Fig. 6). A meta-analysis found sensitivity and specificity of CT for the detection of periannular complications to be 88% and 93% respectively, compared to 70 and 96% for TEE [74]. While some studies have found similar sensitivity between the two modalities, particularly as echocardiography has improved, CT provides significantly more detailed anatomical location and extension than TEE [58, 103]. CT and TEE are best used in conjunction; one study found, when compared to the gold standard of operative findings, the combination of TEE and CT was 100% sensitive for the detection of invasive disease compared to only 86% for TEE [75].

## 7. Nuclear Imaging

### 7.1 Endocarditis

The primary role for nuclear cardiac imaging in the assessment of prosthetic aortic valves is the diagnosis of IE in cases for which clinical and echocardiographic criteria are inconclusive [76]. The literature describes high sensitivity and specificity for nuclear imaging for the diagnosis of endocarditis of prosthetic valves, however controversy exists on the extent of tracer uptake that can be seen in non-infected patients, and therefore careful interpretation at expert centers is warranted.

**Fig. 9.**
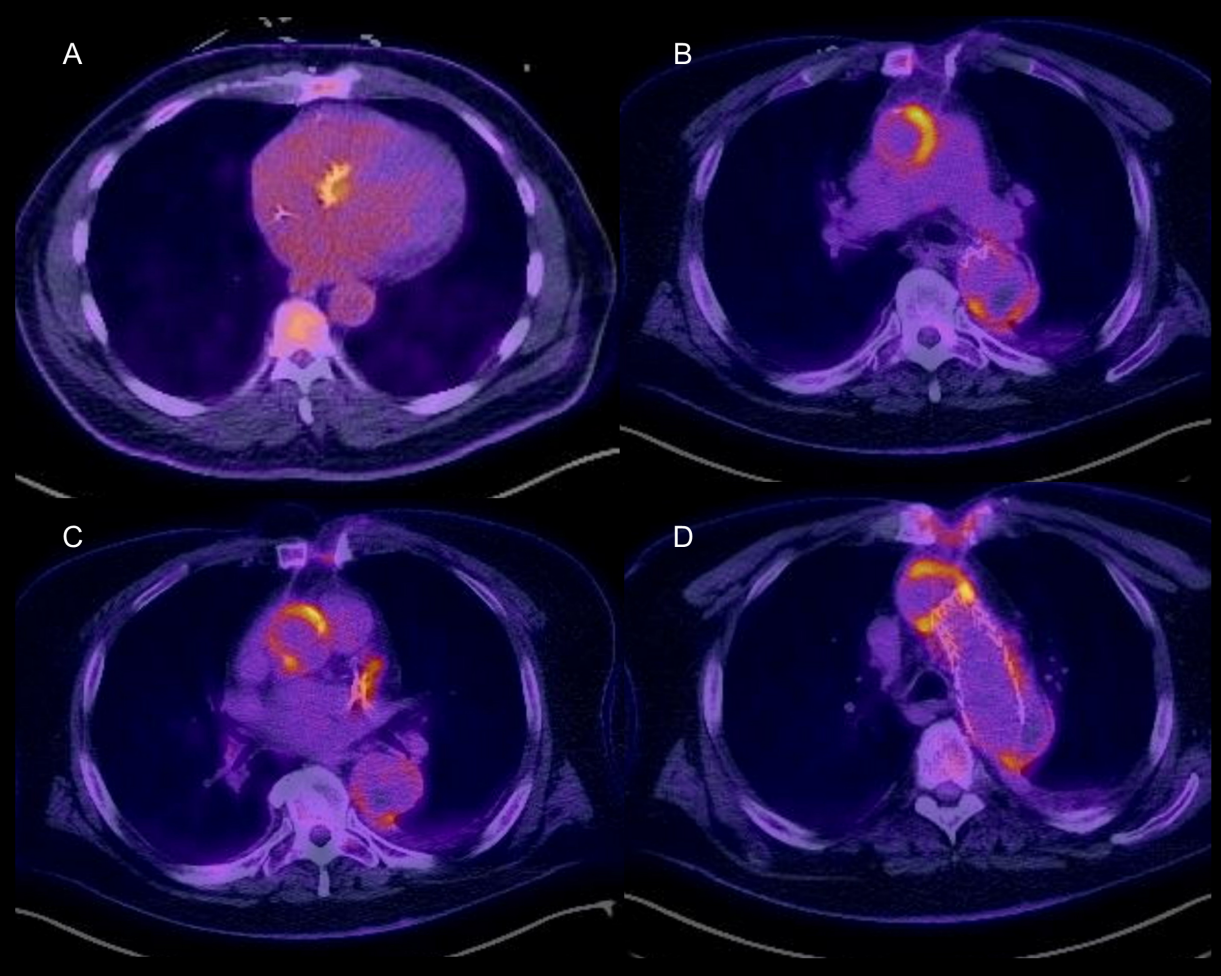
**Assessment of prosthetic valve endocarditis with fluorodeoxyglucose positron emission tomography/computed tomography**. Fluorodeoxyglucose positron emission tomography/computed tomography can aid in the diagnosis of prosthetic aortic valve and aortic graft endocarditis. (A) Patient with streptococcal bacteremia with intense tracer uptake in their bioprosthetic aortic valve. (B–D) Patient with prior Type A dissection, aortic valve repair, ascending aorta and total arch repair with persistent enterococcus faecalis bacteremia. There is intense tracer uptake in the graft.

Two nuclear imaging modalities exist for the detection of prosthetic valve endocarditis: F-18 Fluorodeoxyglucose positron emission tomography/computed tomography (FDG PET/CT) and white blood cell single photon emission computed tomography (WBC SPECT) [104]. FDG-PET/CT has been reported to have high sensitivity (86–97%) and specificity (84%) for prosthetic valve endocarditis (Fig. 9) [105]. A study assessing its additive value to echocardiography found the addition of FDG PET/CT reclassified 90% of patients with “possible” endocarditis by the Duke criteria and provided definitive diagnosis in 95% [106]. It is important to note, the high sensitivity for endocarditis by FDG PET/CT applies only to prosthetic valve endocarditis; FDG PET/CT has low sensitivity (22–68%) in native valve endocarditis [107].

Alternatively, WBC SPECT has higher specificity than PET/CT, up to 100% in some studies, however suffers from poorer sensitivity, with some studies finding it as low as 64% [105, 108]. Differences in sensitivity and specificity are likely explained by the diagnostic mechanism of each modality; the migration of leukocytes, as opposed to simply glucose uptake, is thought to be more specific to infection versus sterile inflammation [108]. Due to a higher sensitivity, FDG PET/CT is the preferred nuclear modality in the most recent ESC guidelines, with a class I indication to confirm the diagnosis of infectious endocarditis. WBC SPECT has a class IIa indication and should be reserved for when FDG PET/CT is unavailable [76].

Nuclear imaging for endocarditis has an additional benefit of identifying other sites of infection. Many patients who undergo aortic valve replacement will also undergo aortic root and ascending aortic replacement. FDG PET/CT has both high sensitivity and specificity for the detection of infected grafts, with one study finding the sensitivity and specificity to be 93% and 91% respectively (Fig. 9) [109, 110]. Additionally, by expanding the field of view beyond the chest to the whole body, FDG PET/CT allows for the detection of other metastatic sites of infection. This often allows for the detection of the precipitating source of the infection, as well as for identifying other abscesses throughout the body that may need surgical drainage to properly achieve source control [108]. Brain and whole-body imaging with FDG PET/CT is a class I indication in the ESC endocarditis guidelines in equivocal cases by Duke criteria or to detect peripheral lesions [76].

The extent of “normal” FDG uptake on a prosthetic valve and the optimal timing from implant to imaging remains controversial. Traditionally, FDG PET/CT has not been recommended in the first three months after implantation, as uptake in the valve is more likely related to resolving surgical inflammation. The ESC guidelines recommend against FDG PET/CT in this early post-operative setting, however this recommendation lacks robust data [111].

Recent studies have suggested that FDG uptake around the prosthesis can persistent well past 3 months. Mathieu *et al*. [112] assessed the uptake of FDG by prosthetic valves in patients undergoing FDG PET/CT for non-cardiac reasons and found the majority of prosthetic valves had some degree of low-level FDG uptake (>90%); there was no significant differences between uptake <3 months (93%) and those >3 months (85%). Roque *et al*. [111] subsequently studied this question prospectively, assessing degree of FDG uptake at 1 month, 6 months, and 12 months after surgical implantation and found diffuse, homogenous uptake in 76% of valves, with little change over the course of the study. Conversely, given how common FDG uptake is, the absence of FDG uptake is thought to have excellent negative predictive value [113]. Due to these findings, experts have proposed diagnostic criteria that integrate qualitative findings, such as pattern and intensity of uptake, quantitative criteria with standardized uptake values, and the degree of peripheral findings suggestive of endocarditis [111]. For these reasons, FDG PET/CT to detect prosthetic valve endocarditis is best used at expert centers with experienced imagers.

**Fig. 10.**
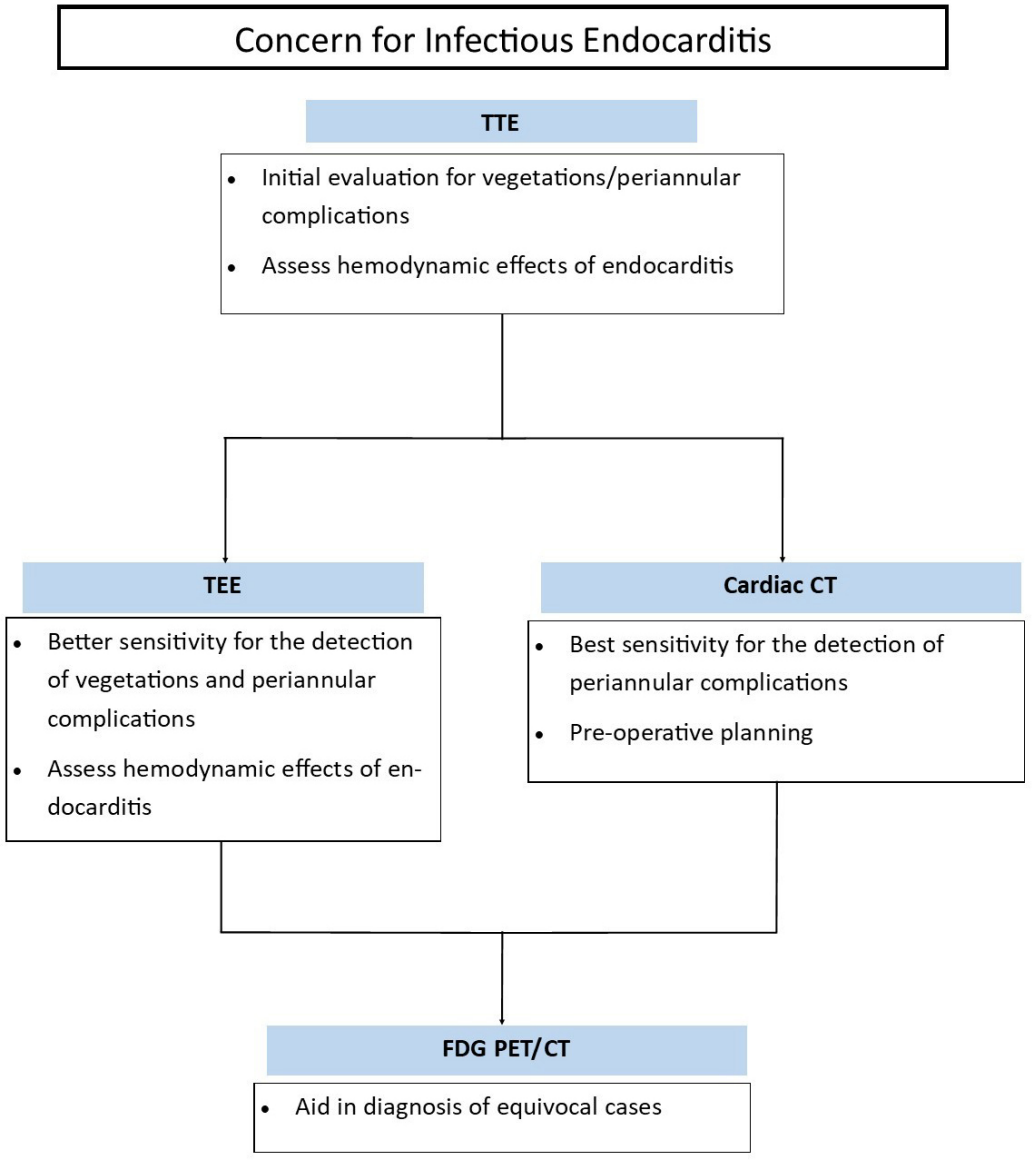
**Common clinical indications for multimodality imaging for the assessment of prosthetic valve endocarditis**. TEE, transesophageal echocardiography; TTE, transthoracic echocardiography; CT, computed tomography; FDG PET/CT, F-18 fluorodeoxyglucose positron emission tomography/computed tomography.

The normal pattern of FDG uptake after TAVR is not yet well-described [57]. Theoretically, there should be less post-operative inflammation after transcatheter valves compared to surgical implants. A small study assessing FDG uptake 3 months after TAVR found the majority of valves did not exhibit significant FDG uptake beyond the degree seen in normal pulmonary parenchyma; there were no significant differences seen between balloon- and self-expanding valves [114]. Defining the normal uptake in TAVR valves, particularly early after implant, is vital as the majority of transcatheter infections occur in the early period after implantation [57]. Fig. 10 shows common clinical indications for multimodality imaging in the setting of IE.

### 7.2 Predicting Prosthetic Valve Degeneration

18F-fluoride (F-18) PET/CT has shown promising use to predict the development of valve degeneration. In a small, prospective study, the presence of F-18 on PET/CT was more predictive of the deterioration of prosthetic valves than cardiac CT. Additionally, many patients who went on to develop valve dysfunction had a normal CT, but abnormal uptake on F-18 PET [115]. This technique is currently pre-clinical, as there have been no formal recommendations on how F-18 PET/CT should be incorporated into clinical practice. This technique may have an investigative role in assessing the durability of novel prosthetic valves or in the development and testing of novel treatments for prosthetic valve disease.

## 8. Cardiac Magnetic Resononance Imaging

The role of CMR in prosthetic valve dysfunction is similar to its role in native valve disease. Firstly, it is important to note that CMR is feasible in patients with prior sternotomy, as sternotomy wires are safe to image [116]. In patients with prosthetic aortic valves, there can be significant artifact at the aortic root which may limit interpretation, but the extent to which this artifact precludes accurate diagnosis often depends on valve characteristics, such as mechanical versus bioprosthetic, SAVR vs TAVR, and the extent and material of the surgical frame [4]. Mechanical valves, for example, will cause significantly more intense and extensive artifact than bioprosthetic valves.

Despite these limitations, quantification of forward and reversed aortic flow in the ascending and descending aorta are often reliable and can give an accurate assessment of aortic regurgitation severity in both valvular and paravalvular regurgitation [117, 118, 119]. Regurgitant fractions are often higher on TEE compared to CMR and therefore different cutoffs should likely be used to denote severe regurgitation [117]. CMR can also measure peak velocity and gradients through aortic valves, however, in native valves, velocities by CMR are often under-estimated compared to TTE [120]. Comparisons of aortic valve velocities by CMR and TTE have not been studied in prosthetic valves. CMR remains the gold-standard for quantifying left ventricular size and function, which may play a significant role in the timing of surgery for prosthetic valves with significant regurgitation [2, 52].

Several measures of tissue characterization by CMR have shown prognostic implications for native AS. The presence of late-gadolinium enhancement, elevated T1 times, and increased extracellular volume have all been associated with poor prognosis and less positive remodeling after aortic valve replacement [121, 122, 123]. These changes in myocardial kinetics likely have similar prognostic significance in patients with prosthetic valve stenosis, although they have not been formally evaluated.

## 9. Artificial Intelligence/Machine Learning

Artificial intelligence/machine learning (AI/ML) is rapidly becoming an integral part of cardiac imaging, with recent strides in ECG, echocardiography, cardiac CT, and CMR [124]. Aortic stenosis has drawn particular interest, with studies describing algorithms that can screen for aortic stenosis using ECG, monitor progression of aortic stenosis with TTE, and aid the diagnosis using multimodality imaging [125, 126, 127, 128, 129]. AI/ML algorithms have also shown promise in the automation of pre-procedural planning by CT in TAVR [130]. However, most current studies have exclusively evaluated the use of AI/ML in native aortic valve disease; these studies currently cannot be generalized to include the evaluation of prosthetic aortic valve disease [124, 131].

An AI/ML algorithm from a recent study by Godefroy *et al*. [132] showed promise in the diagnosis of prosthetic valve endocarditis via FDG PET/CT, however the study was limited by small sample size (n = 108). A major limitation in all AI/ML-based imaging studies, in particular of prosthetic valve disease, is the lack of large, high-quality data sets needed for both training and testing AI/ML algorithms [133]. Furthermore, prospective trials are then needed to assess the incremental benefit over current practice with the application of AI/ML algorithms.

## 10. Conclusions

With the introduction of TAVR, particularly its expansion to low-risk populations, the number and complexity of patients with prosthetic aortic valves is increasing. Fortunately, the treatment options available to patients with prosthetic aortic dysfunction is also increasing. As a result, there is greater emphasis on accurate diagnosis of prosthetic valve pathology. Multimodality imaging has become fundamental to the care of these patients. Each type of imaging has its strengths and weaknesses and therefore the contemporary care of these patients, whether by a general cardiologist, cardiac imager, interventional cardiologist, or cardiac surgeon, requires the ability to integrate data from these different modalities. It is through multimodality imaging that we can formulate the best treatment plan for our patients.
